# What does a varicocele do to a man's fertility? There is much more than meets the eye

**DOI:** 10.1590/S1677-5538.IBJU.2019.0827.1

**Published:** 2021-02-03

**Authors:** Sheena E.M. Lewis, Sandro C. Esteves

**Affiliations:** 1 Queens University Belfast United Kingdom Queens University Belfast, Northern Ireland, United Kingdom; 2 Examenlab Ltd. Weavers Court United Kingdom Examenlab Ltd., Weavers Court, Belfast, Northern Ireland, United Kingdom; 3 ANDROFERT Andrology and Human Reproduction Clinic Campinas SP Brasil ANDROFERT, Andrology and Human Reproduction Clinic, Campinas, SP, Brasil; 4 Universidade Estadual de Campinas -UNICAMP Divisão de Urologia Departamento de Cirurgia Campinas SP Brasil Departamento de Cirurgia, Divisão de Urologia, Universidade Estadual de Campinas -UNICAMP, Campinas, SP, Brasil; 5 Aarhus University Faculty of Health Aarhus Denmark Faculty of Health, Aarhus University, Aarhus, Denmark

## COMMENT

Varicoceles are so common in infertile men they are believed to be a leading cause of male infertility (1). Up to 40% of men with primary infertility have varicoceles, and this figure increases to a staggering 80% of men with secondary infertility (2).

Despite this, clinical examinations are seldom performed on men attending for infertility investigations, and hence neither detection of a varicocele nor its treatment is routinely included in the male workup. Although there is strong evidence that varicocele repair improves semen parameters (reviewed by Tiseo et al. (3)), there is a poor association of a conventional semen analysis with either male infertility diagnosis or with successful ART (reviewed by (4, 5)). This has led to a dismissal of the need to improve sperm parameters quality prior to assisted reproductive treatment (ART), especially when intracytoplasmic sperm injection (ICSI) can be used as a faster alternative to overcome the problem (6, 7).

In a recent study, Jeremias et al. have sought to identify the impact of varicocele on a more critical sperm health marker than a semen analysis; its DNA (8). Sperm DNA provides the genetic template for a man's offspring, and its quality impacts significantly on children's short- and long-term health. In summary, this latest data supports that of previous studies indicating that varicocele is strongly associated with DNA damage. However, Jeremias and co-workers have drilled deeper and by using the sensitive and versatile Comet assay (9), at both alkaline and neutral pH, they have been able to detect total DNA damage and double-stranded breaks separately. They have also included a base excision repair enzyme -formamidopyrimidine DNA glycosylase (FPG)– to convert 8-OH-guanine and 8-OH-20-deoxyguanosine adducts into further single-strand breaks as a measure of damage induced explicitly by oxidative stress. Using this approach, the authors added to our knowledge as they showed a markedly elevated double-stranded, oxidatively induced, and total DNA damage in sperm of men with grade 2 or 3 uni- or bilateral varicoceles.

To the best of our knowledge, this is the first study detailing the type of DNA damage in men with clinically significant varicoceles. It provides direct evidence that in men with varicocele DNA damage is oxidatively induced. Equally important, it shows that clinical varicocele causes both single-strand DNA breaks and double-strand DNA breaks; the latter being more severe and difficult to repair by oocyte repair mechanisms. Another novel aspect of their work relates to the inclusion of normozoospermic men -those who have conventional semen parameters within normal ranges. Again, to our knowledge, this is the first study to report that normozoospermic men with clinically significant varicoceles can cause sperm DNA damage even though conventional semen parameters do not show evident abnormalities.

Jeremias and co-workers’ study has an important clinical impact as a plethora of studies over the past decade have reported associations between sperm DNA damage inducers, such as ageing and smoking, and an increased risk of childhood cancers and neurological defects in offspring (10–12). It also has critical clinical implications for counselling, diagnosis, and treatment as varicocele repair is not generally endorsed unless conventional semen parameters are abnormal (13). The benefits of varicocele repair on improving the chance of either natural conception or successful ART are still sufficiently controversial to prevent its routine inclusion in fertility clinics. The current UK National Institute of Health and Care Excellence guidance is that men should not be offered surgery for varicoceles as a form of fertility treatment because it does not improve pregnancy rates (14).

In contrast, several recent reviews have supported the role of varicocele repair in improving male fertility specifically by its improvement to sperm DNA quality (15–18). Additionally, an evidence-based algorithm was recently published by the Society of Translational Medicine to guide urologists on the indications of sperm DNA fragmentation testing, and improvement of fertility by varicocelectomy (19). Again, the mechanism by which this improvement occurred was by a reduction in sperm DNA damage. According to the guidelines above, varicocele repair should be offered as part of the treatment option for all male partners of infertile couples presenting with palpable varicoceles and sperm DNA damage, regardless of the conventional semen analysis results. The study by Jeremias and co-workers provides further evidence supporting the adverse impact of varicocele on sperm DNA of normozoospermic men, and the need for including sperm DNA fragmentation testing in the routine evaluation of men with varicocele seeking fertility (4, 6, 20).

A further impediment to providing a full male investigation is the lack of uro-andrologists on fertility clinic teams. This is commonly the case in the UK, although less common in the USA and Brazil. Most UK fertility clinics are staffed by obstetricians, so they are female focussed. Traditionally, these clinicians have little training in the causes of male infertility and hence in the male partner. This required urgent redress as a matter of best clinical practice.

A final consideration for the inclusion of varicocele in the male fertility workup is cost-effectiveness. Cost-benefit analyses were reviewed by Yan and co-workers in 2019, concluding that surgical repair of the varicocele is the most cost-effective primary treatment compared to any form of ART (21).

For all these reasons, we ask readers: ‘Is it time to review our traditional male workups based on the latest scientific and economic evidence and provide men with an optimal fertility pathway?
